# Supplementary material for: End Sequence Analysis Toolkit (ESAT) expands the extractable information from single-cell RNA-seq data
Source: Genome Res. 2016 Oct;26(10):1397–410. doi: 10.1101/gr.207902.116 (PMC5052061; doi:10.1101/gr.207902.116)
Supplement: Supplemental Material [file supp_gr.207902.116_Supp_Materials.tar.gz › ESAT-esat_v0.1/doc/html/classjaligner_1_1_cell.html]

ESAT: jaligner.Cell Class Reference


ESAT


- Main Page
- Related Pages
- Packages
- Classes
- Files

- Class List
- Class Index
- Class Hierarchy
- Class Members

All Classes Namespaces Files Functions Variables Enumerator Pages

- jaligner
- Cell

Public Member Functions |
List of all members

jaligner.Cell Class Reference

|  |  |
| --- | --- |
| Public Member Functions | |
|  | Cell () |
|  | |
| int | getCol () |
|  | |
| void | setCol (int col) |
|  | |
| int | getRow () |
|  | |
| void | setRow (int row) |
|  | |
| float | getScore () |
|  | |
| void | setScore (float score) |
|  | |
| void | set (int row, int col, float score) |
|  | |

## Detailed Description

A cell in a similarity matrix, to hold row, column and score.

Author
:   Ahmed Moustafa (ahmed.nosp@m.@use.nosp@m.rs.sf.nosp@m..net)

## Constructor & Destructor Documentation

|  |  |  |  |  |
| --- | --- | --- | --- | --- |
| jaligner.Cell.Cell | ( |  | ) |  |

Constructor

## Member Function Documentation

|  |  |  |  |  |
| --- | --- | --- | --- | --- |
| int jaligner.Cell.getCol | ( |  | ) |  |

Returns
:   Returns the col.

|  |  |  |  |  |
| --- | --- | --- | --- | --- |
| int jaligner.Cell.getRow | ( |  | ) |  |

Returns
:   Returns the row.

|  |  |  |  |  |
| --- | --- | --- | --- | --- |
| float jaligner.Cell.getScore | ( |  | ) |  |

Returns
:   Returns the score.

|  |  |  |  |
| --- | --- | --- | --- |
| void jaligner.Cell.set | ( | int | *row*, |
|  |  | int | *col*, |
|  |  | float | *score* |
|  | ) |  |  |

Sets the row, column and score of the cell.

Parameters
:   |  |  |
    | --- | --- |
    | row | The row to set. |
    | col | The col to set. |
    | score | The score to set. |

|  |  |  |  |  |  |
| --- | --- | --- | --- | --- | --- |
| void jaligner.Cell.setCol | ( | int | *col* | ) |  |

Parameters
:   |  |  |
    | --- | --- |
    | col | The col to set. |

|  |  |  |  |  |  |
| --- | --- | --- | --- | --- | --- |
| void jaligner.Cell.setRow | ( | int | *row* | ) |  |

Parameters
:   |  |  |
    | --- | --- |
    | row | The row to set. |

|  |  |  |  |  |  |
| --- | --- | --- | --- | --- | --- |
| void jaligner.Cell.setScore | ( | float | *score* | ) |  |

Parameters
:   |  |  |
    | --- | --- |
    | score | The score to set. |

---

The documentation for this class was generated from the following file:

- java/jaligner/Cell.java


---

Generated on Fri Aug 15 2014 15:11:05 for ESAT by  

 1.8.7
